# The beneficial effects of meditation: contribution of the anterior cingulate and locus coeruleus

**DOI:** 10.3389/fpsyg.2013.00731

**Published:** 2013-10-16

**Authors:** Nancy A. Craigmyle

**Affiliations:** Gurdjieff Foundation of CaliforniaSan Francisco, CA, USA

**Keywords:** meditation, salience detecting, anterior cingulate, locus coeruleus, norepinephrine

## Abstract

During functional magnetic resonance imaging studies of meditation the cortical salience detecting and executive networks become active during “awareness of mind wandering,” “shifting,” and “sustained attention.” The anterior cingulate (AC) is activated during “awareness of mind wandering.” The AC modulates both the peripheral sympathetic nervous system (SNS) and the central locus coeruleus (LC) norepinephrine systems, which form the principal neuromodulatory system, regulating in multiple ways both neuronal and non-neuronal cells to maximize adaptation in changing environments. The LC is the primary source of central norepinephrine (C-NE) and nearly the exclusive source of cortical norepinephrine. Normally activated by novel or salient stimuli, the AC initially inhibits the SNS reflexively, lowering peripheral norepinephrine and activates the LC, increasing C-NE. Moderate levels of C-NE enhance working memory through alpha 2 adrenergic receptors, while higher levels of C-NE, acting on alpha 1 and beta receptors, enhance other executive network functions such as the stopping of ongoing behavior, attentional set-shifting, and sustained attention. The actions of the AC on both the central and peripheral noradrenergic systems are implicated in the beneficial effects of meditation. This paper will explore some of the known functions and interrelationships of the AC, SNS, and LC with respect to their possible relevance to meditation.

## INTRODUCTION

During a recent functional magnetic resonance imaging (fMRI) study of focused mindfulness meditation the cortical salience detecting and executive networks, were shown to become active during “awareness of mind wandering,” “shifting,” and “sustained attention,” while the default mode network (DMN) was active during “mind wandering” (Hasenkamp et al., 2012). This paper will primarily address mindfulness meditation, but awareness of mind wandering, shifting, and sustained attention could be expected to be involved, at least initially, in all forms of meditation.

The salience detecting and executive networks include the anterior insula, anterior cingulate (AC), anterior inferior parietal, dorsolateral prefrontal cortex (DLPFC), and anterior medial prefrontal cortex (Sturm et al., 1999; Sridharan et al., 2008; Vincent et al., 2008). These areas are essentially the same as those of the highly interconnected frontoparietocingulate (FPC) system thought to represent the cortical component of the orienting system (Halgren et al., 1998) and to underlie intrinsic alertness (Sturm et al., 1999; Kiehl et al., 2001, 2005; Dien et al., 2003; Calhoun et al., 2006).

The AC is a major component of the medial prefrontal cortex (mPFC). In much of the relevant literature, the mPFC is roughly divided into two subregions, relative to the genu of the corpus callosum: the dorsal medial prefrontal cortex (dmPFC) and the ventral medial prefrontal cortex (vmPFC) (Kim et al., 2011a).

Broadly defined, the dmPFC includes areas of the salience detecting/executive FPC network including the dorsal AC [BA24 and BA32], the anterior medial frontal cortex [BA10], and the DLPFC [BA9/46]. The areas of the medial frontal gyrus encompassing the dorsal attention network (DAN), including the pre-supplementary motor area (pre-SMA) and frontal eye fields [BA8], and the supplementary motor area (SMA) [BA6] are also a part of the dmPFC. The vmPFC includes the subgenual AC area [BA25], the vmPFC [ventral portions of BA32 and BA10], and the medial orbitofrontal cortex [BA11 and BA12], areas associated with the DMN and the amygdala (AM).

The salience detecting/executive FPC network shifts between and relates to both the externally directed DAN, which receives the stimuli of the present moment from the external environment, and to the internally directed hippocampal–cortical memory system, a part of the DMN (Sridharan et al., 2008; Vincent et al., 2008).

The salience detecting anterior insula and AC of the FPC network also receive the interoceptive information from within the organism, which underlies the sense of oneself and of one’s emotions (Craig, 2009). The interoceptive information is carried to the cortical anterior insula and AC, as well as to the somatosensory cortex, ventrolateral medulla (VLM), and the locus coeruleus (LC) (Craig, 1992) from the peripheral noradrenergic sympathetic nervous system (SNS) via its ascending lamina 1 spinothalamocortical tract (STT) (Craig, 2009).

In turn, the dorsal AC (Verberne, 1996; Verberne et al., 1997; Verberne and Owens, 1998; Viltart et al., 2003) and the insula (Hardy, 1994; Ter Horst et al., 1996) modulate activity in the SNS via the rostral VLM. Inhibition of the rostral VLM causes a reflex fall in SNS nerve activity, resulting in decreased peripheral norepinephrine (P-NE), and decreased blood pressure (Standish et al., 1995).

The more ventral, emotion associated, portions of the AC [BA 25 and BA32] also project to the parasympathetic vagal nuclei including the dorsal motor nucleus (DM), the nucleus of the solitary tract (NTS), and to both the periambiguus and core areas of the nucleus ambiguus (NA) (Hurley et al., 1991; Buchanan et al., 1994).

The NA contains primary source nuclei for the cardiopulmonary branch of the vagus and has myelinated axons thought to rapidly modulate the function of the heart and lungs (Standish et al., 1995). Some neurons in the NA simultaneously innervate both the adrenal gland and the stellate sympathetic ganglion (Jansen et al., 1995) through which it can rapidly modulate the SNS.

The NA is also closely linked to the rapid expression and regulation of emotional state (Porges, 1995; Tonhajzerova et al., 2013). A withdrawal of the cardiopulmonary vagal efferent outflow from the NA is seen during both acute and chronic stress, which, in healthy individuals, is accompanied by increased sympathetic tone (Porges, 1995), by increased levels of P-NE.

Rapid autonomic changes, including cardiovascular and respiratory changes, such as respiratory rate, respiratory sinus arrhythmia, and heart rate variability (Reyes Del Paso et al., 2013; Tonhajzerova et al., 2013), are thought to occur by way of the parasympathetic NA (Porges, 1995; Standish et al., 1995; Wong et al., 2007; Tonhajzerova et al., 2013), in conjunction with the rostral and caudal VLM (Standish et al., 1995).

Central and autonomic interactions altered by short-term meditation suggest control of parasympathetic activity by the AC [BA25 and BA32] (Tang et al., 2009). This control of parasympathetic activity and the resultant inhibition of the SNS during meditation may occur by way of the AC [BA 25 and BA 32] projections to the NA (Hurley et al., 1991; Buchanan et al., 1994). During meditation greater parasympathetic activity is observed in the lower heart rate and skin conductance, increased belly respiratory amplitude, decreased chest respiration rate and increased high-frequency heart rate variability (HF-HRV) (Tang et al., 2009).

The AC also directly modulates the central norepinephrine (C-NE) levels by increasing activity in the LC (Jodo et al., 1998; Rajkowski et al., 2000), the principal central noradrenergic nucleus.

The AC is normally activated by novel or significant stimuli (Aston-Jones and Cohen, 2005). In turn, the AC activates the LC (Jodo et al., 1998; Rajkowski et al., 2000) exerting a tonic influence (Jodo et al., 1998) on LC baseline firing, while LC burst firing to stimuli is thought to reflect AC decisions following the stimuli (Clayton et al., 2004; Nieuwenhuis et al., 2005a).

The LC projects throughout the brain, is the principal source of C-NE in the thalamus (McCormick et al., 1991; Robertson et al., 2013), and the only known source of C-NE in the hippocampus and cortex, with the exception of a recently described projection to the insular and orbital prefrontal cortex of C-NE (r4-derived) neurons from the subcoeruleus nuclei and from the caudal portions of the C2/A2 and C1/A1 sympathetic nuclei of the medulla thought to receive visceral sensory, interoceptive input from the peripheral nervous system (Robertson et al., 2013).

The LC acts both synaptically and by volume transmission (O’Donnell et al., 2012) on its target neurons in the brain and differentially effects the different types of neurons found within each section of the areas to which it projects (Chandler and Waterhouse, 2012). C-NE participates in the rapid modulation of cortical circuits and cellular energy metabolism, and on a slower time scale in neuroplasticity and inflammation (O’Donnell et al., 2012).

Silent only during rapid eye movement (REM) sleep (Hobson and Stickgold, 1994), increases in LC baseline firing progressively increases wakefulness, cortical arousal (Berridge et al., 2012a), the neuronal signal to noise ratio (Aston-Jones et al., 1999), and receptivity to the sensory signals of the present moment (Foote et al., 1991). Although the LC can be activated by stress (Aston-Jones and Cohen, 2005), this wakeful receptivity and cortical arousal are not the same as the SNS arousal under stress; there is an inverse relationship between cortical arousal and peripheral sympathetic arousal (Nagai et al., 2004a, b, 2009; Duschek et al., 2007, 2013).

The integrated P-NE and C-NE systems form the principal neuromodulatory system, a homeostatic system regulating in multiple ways the activity of both neuronal and non-neuronal (astrocytes and microglial) cells (O’Donnell et al., 2012) to adapt the state of both the body and the brain for optimal functioning in changing environments.

As a part of the salience detecting/executive FPC network, the AC is in a position to integrate the information (Wang et al., 2005; Vincent et al., 2008) concerning the state of the external, the internal (Vincent et al., 2008), and the interoceptive environments in the present moment. By rapidly modulating the activity levels of both the principal NE systems, the AC is in a position to adapt the state of the whole organism to optimize attention and behavior as changes are detected in any of these environments.

The LC is thought to optimize attention and behavior in changing environments (Aston-Jones and Cohen, 2005). Activation of the AC is accompanied by a widespread coactivation of other areas of the brain (Wang et al., 2005). A number of fMRI studies of the cortical orienting response of intrinsic alertness have indicated that activity in the salience detecting/executive FPC network is accompanied by an activation of the LC (Sturm et al., 1999; Kiehl et al., 2001, 2005; Dien et al., 2003; Calhoun et al., 2006). This activation of the LC is associated with the widespread coactivation of other areas until the event-encoding cycle ends (Halgren et al., 1998). As the cycle ends activity decreases in the initial areas associated with orienting, while effective connectivity between relevant cortical areas increases (Büchel et al., 1999; McIntosh et al., 1999).

All the areas of the brain activated by the intentional, impartial, sustained attention of meditation are of significant, interrelated importance, including the insula, the other cortical salience detecting network area (Craig, 2009; Menon and Uddin, 2010). One of the largest activations at the moment of awareness of mind wandering, however, is seen in the AC (Hasenkamp et al., 2012). In this paper we will briefly explore some of the known functions and interrelationships of the AC, the SNS, and the LC with respect to their possible relevance to the process of meditation. The LC core and the subcoerulear (Westlund and Coulter, 1980) or pericerulear (Shipley et al., 1996; Aston-Jones et al., 2004) areas will be, here, treated as one, except where specifically mentioned.

## UNCONSCIOUS DETERMINANTS OF CONSCIOUS DECISIONS

Immediately prior to the conscious awareness of voluntary, self-determined conscious decisions there is a consistent decrease in heart rate (Tallon-Baudry, 2012). Studies of the unconscious determinants of voluntary conscious decisions (Soon et al., 2008; Bode et al., 2011; Fried et al., 2011; Kreiman, 2012) have shown that the anterior medial prefrontal cortex, also called the rostral AC [BA10] (Soon et al., 2008; Bode et al., 2011; Fried et al., 2011), the SMA, pre-SMA, and the dorsal AC are active prior to conscious awareness (Fried et al., 2011; Kreiman, 2012). The SMA, pre-SMA, and AC are known to inhibit the SNS via the rostral VLM (Viltart et al., 2003) causing a reflex fall in sympathetic nerve activity and blood pressure (Standish et al., 1995). The ventral AC (Hurley et al., 1991; Buchanan et al., 1994) and the pre-SMA (38 Buchanan et al., 1994) can also modulate the SNS through their projections to the parasympathetic vagal nuclei, while the anterior medial prefrontal cortex [BA10], along with ventral portions of BA 32, has been found to covary inversely with skin conductance (Critchley et al., 2000; Nagai et al., 2004c) and directly with HF-HRV (Lane et al., 2009).

## HEART RATE VARIABILITY

Ventral AC activation has been found in fMRI studies to be significantly correlated with HF-HRV suggesting AC control of parasympathetic autonomic activity (Tang et al., 2009). The pregenual mPFC [BA10/BA32], right superior frontal gyrus [BA10/46)], and left and right parietal cortex [BA40] of the salience detecting/executive FPC cortical orienting system are also positively correlated with HF-HRV, indicating that as activity increases in these cortical areas the vagal breaking action on the heart also increases, reflecting a vagal inhibition of sympathetic influences (Lane et al., 2009).

As mentioned, rapid autonomic changes, including cardiovascular and respiratory changes, such as respiratory rate, respiratory sinus arrhythmia, and heart rate variability (Reyes Del Paso et al., 2013; Tonhajzerova et al., 2013), are thought to occur by way of the parasympathetic NA (Porges, 1995; Standish et al., 1995; Wong et al., 2007; Tonhajzerova et al., 2013), in conjunction with the rostral and caudal VLM (Standish et al., 1995).

Central and autonomic interactions altered by short-term meditation suggest control of parasympathetic activity by the ventral AC [BA25 and BA32] (Tang et al., 2009). This control of parasympathetic activity and the resultant inhibition of the SNS during meditation may occur by way of the AC [BA 25 and BA 32] projections to the NA (Hurley et al., 1991; Buchanan et al., 1994). During meditation greater parasympathetic activity is observed in the lower heart rate and skin conductance, increased belly respiratory amplitude, decreased chest respiration rate, and increased HF-HRV (Tang et al., 2009).

The decreases in respiratory rate, whether spontaneous or intentional, and the increases in HF-HRV that occur during meditation may all reflect the same vagal inhibition of sympathetic influences.

The positive correlation of the FPC cortical areas with HF-HRV also suggests that HF-HRV is low when peripheral arousal is high (Lane et al., 2009). As mentioned, a withdrawal of vagal efferent outflow from the NA is seen during both acute and chronic stress, accompanied, in healthy individuals, by increased sympathetic tone (Porges, 1995; Tonhajzerova et al., 2013). The influence of FPC cortical activity on the vagal nuclei may also contribute to the inverse correlation between cortical and peripheral arousal (Nagai et al., 2004a, b, 2009).

## THALAMUS AND CORTEX

The C-NE of the LC has potent and long-lasting effects on thalamic and cortical neurons (McCormick et al., 1991; Coull et al., 1999) and is intimately involved in determining both the level of neuronal excitability and the pattern of activity generated by neurons in thalamocortical systems (McCormick et al., 1991). C-NE, for example, was shown, via alpha 2 adrenergic receptors, to increase functional integration and connectivity from the LC to the parietal cortex and from the parietal cortex to the thalamus and frontal cortex, implicating the LC-noradrenergic system in mediating the functional integration of attentional brain systems (Coull et al., 1999).

Increases in C-NE and serotonin in both the thalamic reticular nucleus (RN) and the lateral geniculate nucleus (LGN) shift the thalamic firing pattern from a spontaneous rhythmic bursting mode to a single-spike mode (McCormick and Wang, 1991). These modes of firing are associated, respectively, with inattention, drowsiness, and sleep or with wakefulness and arousal (Steriade and Llinás, 1988).

Stimulation of the LC, simulating LC burst firing, increases neuronal firing in the LGN resulting in a highly discriminable signal in the LGN. During waking, LGN neurons have a single-spike firing mode when sensory information is faithfully relayed from the retina to the cortex and a burst-firing mode when the transfer of this information is degraded (Holdefer and Jacobs, 1994). During REM sleep the transfer of retinal information to the cortex ceases when the LC and the serotonergic dorsal raphe nucleus stop firing (Hobson and Stickgold, 1994).

## EEG WAVES

Large-scale cortical synchrony, which depends on the integrity of corticothalamic feedback, is thought to act predominantly through the RN (Destexhe et al., 1998). Although the fusiform gyrus is implicated in the generation of high amplitude gamma synchrony during meditation (Lutz et al., 2004), it has been suggested that the RN plays a pacemaker function in the genesis of 40-Hz gamma oscillations in the thalamus and cortex during states of focused arousal (Pinault and Deschênes, 1992a). Bilateral lesions of the LC, and local application of the alpha 1 adrenergic antagonist, prazosin, abolished reticular thalamic 40-Hz, gamma firing (Pinault and Deschênes, 1992b), indicating a modulation of this system by the LC.

Data suggests that the AC generates electroencephalographic (EEG) task-related theta waves (Wang et al., 2005). Frontal midline theta rhythm is associated with activation of the parasympathetic nervous system (Tang et al., 2009) and negatively correlated with sympathetic activation (Kubota et al., 2001). The phase of the low-frequency theta rhythm modulates power in the high gamma wave band, with stronger modulation occurring at higher theta amplitudes (Canolty et al., 2006). The task-related increase in theta-phase locking indicates AC theta-phase locking forms part of a large network involving widespread cortical locations, consistent with the widespread coactivation of the AC with other areas as observed with fMRI (Wang et al., 2005). The widespread coactivation has been associated with an activation of the LC (Sturm et al., 1999; Kiehl et al., 2001, 2005; Dien et al., 2003; Calhoun et al., 2006).

There are both direct cholinergic and serotonergic pathways and indirect modulatory LC and AM pathways in EEG activation (Dringenberg and Vanderwolf, 1997, 1998). Increases in LC neuronal activity increases EEG activity in both the hippocampal theta and cortical high-frequency gamma wave bands (Berridge and Foote, 1991, 1996; Berridge, 1998) through its action on the medial septum (Berridge et al., 1996; Smiley et al., 1999).

Alpha waves, on the other hand, are associated with the DMN, which was found to be active during mind wandering in a focused mindfulness meditation study (Hasenkamp et al., 2012). Alpha waves are seen in novice mindfulness meditators, however, a decrease in alpha waves accompanies increased experience in longer term mindfulness meditators (Saggar et al., 2012).

Alpha waves spontaneously occur intermittently during an awake, relaxed, resting state, particularly with closed eyes (Goldman et al., 2002; DiFrancesco et al., 2008), when the vmPFC and DMN are active and they normally stop during salience detecting orienting responses to novel or significant stimuli, or with mental effort (Goldman et al., 2002), when the dmPFC and DAN are active.

Understanding of the origins of the occipital alpha rhythm is incomplete, but a plausible scheme is thought to include a complex interplay between the LGN and RN of the thalamus and the visual cortex (DiFrancesco et al., 2008). The central role of the thalamus in resting state networks is correlated with alpha activity (DiFrancesco et al., 2008) and it has been suggested that the alpha rhythm may be in part generated by the thalamus (Goldman et al., 2002).

In the resting state a decrease in the release of C-NE and serotonin in the thalamus may promote the occurrence of rhythmic oscillations (McCormick and Wang, 1991).

Alpha EEG waves are frequently recorded during studies of transcendental meditation (Travis and Shear, 2010). Beyond the categories of focused and open monitoring meditation, a third meditation category of automatic self-transcending has been proposed to explain the differences in the prevalence of alpha waves (Travis and Shear, 2010).

During mindfulness meditation, increased EEG activity in both theta (Kubota et al., 2001; Cahn and Polich, 2006; Slagter et al., 2009; Tang et al., 2009; Cahn et al., 2013) and gamma (Lutz et al., 2004; Cahn and Polich, 2006; Cahn et al., 2010, 2013) wave lengths have been recorded. The AC and its relationship with the LC may contribute to the changes in theta and gamma waves.

## DECISIONS, EVENT-RELATED POTENTIALS, AND THE ATTENTIONAL BLINK

Anterior cingulate activity elevates LC baseline firing (Jodo et al., 1998), and LC phasic burst responses are thought to reflect AC decisions following novel or significant known stimuli (Clayton et al., 2004; Aston-Jones and Cohen, 2005; Nieuwenhuis et al., 2005a).

The P300 event-related potential (ERP) is thought to index AC activated LC phasic burst responses to stimuli (Murphy et al., 2011) and the LC is hypothesized to play a role in mediating the attentional blink (Nieuwenhuis et al., 2005b) as well as the accompanying pupil dilations (Zylberberg et al., 2012).

Lower (phasic) baseline firing in the LC is associated with large phasic bursts of firing to selectively attended stimuli (Aston-Jones and Cohen, 2005) accompanied by proportionately longer refractory periods, thought to be due to the autoinhibitory alpha 2 adrenergic receptors in the LC (Nieuwenhuis et al., 2005b). In contrast, higher (tonic) baseline firing is associated with more frequent smaller bursts to the various stimuli of the present moment, including low salience “distractor” stimuli (Aston-Jones and Cohen, 2005), followed by shorter refractory periods (Nieuwenhuis et al., 2005b).

In meditators, the P3a ERP amplitude to distractor stimuli is reduced (Cahn and Polich, 2009) and they demonstrate an enhanced receptivity to the second target in an attentional blink paradigm (Slagter et al., 2007, 2009; van Leeuwen et al., 2009). A reduced brain-resource allocation to the first target is hypothesized to underlie the enhanced receptivity to the second target (Slagter et al., 2007, 2009) and the shorter refractory periods associated with the reduced phasic burst release of C-NE by the LC are thought to contribute to the enhanced availability to the second target (Nieuwenhuis et al., 2005b).

The increased activation of the AC during meditation would be expected to cause higher tonic LC baseline firing in meditators, while the associated more frequent smaller bursts to various stimuli and their shorter refractory periods may underlie the meditators’ enhanced receptivity to the second target as suggested by the existing hypotheses (Nieuwenhuis et al., 2005b; Slagter et al., 2007, 2009).

## PUPIL DILATION

Pupil dilation, under constant illumination, is mediated almost exclusively via C-NE release by the LC (Koss, 1986; Einhäuser et al., 2010). Pupil diameter has been hypothesized to reflect both the tonic and the phasic aspects of LC activity, with large baseline pupil diameter equating with high tonic LC activity (Rajkowski et al., 1993) and brief increases in diameter with phasic activity following stimuli.

Pupil dilation following stimuli reflects AC decisions (Aston-Jones and Cohen, 2005; Einhäuser et al., 2010; Preuschoff et al., 2011). The dilation further reflects perceptual selection and predicts subsequent stability in perceptual rivalry (Einhäuser et al., 2008). During meditation, long-term meditators demonstrate enhanced pupil dilation to stimuli (Carter et al., 2005; Brefczynski-Lewis et al., 2007) and, in a study of binocular vision in long-term meditators, their larger pupil dilation was predictive of their subsequent longer durations between binocular shifts (Carter et al., 2005). This again suggests enhanced LC activation in long-term meditators. Humans with dopamine beta hydroxylase deficiency have a complete absence of C-NE and P-NE and they exhibit an abnormally small or absent task-evoked pupil dilation (Jepma et al., 2011).

## ATTENTIONAL SET-SHIFTING, SUSTAINED ATTENTION, AND THE STOP OF ON GOING BEHAVIOR

In the cortex, relatively low, moderate levels of C-NE act on the alpha 2 adrenergic receptors to enhance working memory in the DLPFC in an inverted U shaped manner (Arnsten, 2011). Higher levels of C-NE are required to act on alpha 1 adrenergic receptors in order to enhance the executive network functions of attentional set-shifting and sustained attention (Berridge and Arnsten, 2012; Berridge et al., 2012b). Improvement was blocked by the alpha 1-antagonist prazosin (Berridge et al., 2012b). Higher levels of C-NE, from the activation of the LC by the AC, may contribute to the attentional shifting and sustained attention observed during meditation.

During the salience detecting orienting response there is an initial inhibition of ongoing behavior (Foote et al., 1980, 1991; Rasmussen et al., 1986), a stop, including a cessation of movement (Ball et al., 1999).

A stop of on going behavior is required for attentional set-shifting, as seen in meditators. As with attentional set-shifting and sustained attention, the stopping of on going behavior is improved by higher levels of C-NE acting on cortical alpha 1 and beta adrenergic receptors (Lapiz and Morilak, 2006; Berridge and Arnsten, 2012; Berridge et al., 2012b), while more moderate levels, acting on alpha 2-adrenoreceptors, improve working memory in the DLPFC (Arnsten, 2011). Elevated C-NE in the “prelimbic” AC [BA32] improves the stopping of ongoing behavior (Bari et al., 2010).

## TASK DIFFICULTY

The synaptic specializations of the dorsal AC area BA32 indicate it has complementary roles, potentially enhancing the inhibition of spontaneous firing in the working memory DLPFC area [BA46] and strengthening excitation in the anterior medial prefrontal cortex [BA10], enhancing the capacity for more difficult, complex multi task operations (Medalla and Barbas, 2010). LC activity, similarly, increases with task difficulty (Rajkowski et al., 2004; Raizada and Poldrack, 2007).

## SMA AND PRE-SMA

The AC has connections to the SMA, which mediates the preparation and initiation of movement (Devinsky et al., 1995). The intermediate SMA has been found to inhibit the primary motor area (M1) until a decision is made by the AC, before releasing it to action (Ball et al., 1999), while the pre-SMA has been shown to switch from habitual automatic to volitionally controlled saccades by inhibiting the habitual, automatic action (Hikosaka and Isoda, 2008). The pre-SMA has also been shown to contribute to the free choice of self-initiated actions (Thimm et al., 2012) and to be more active and functionally correlated with the DLPFC during internally compared to externally guided action planning (Rosenberg-Katz et al., 2012).

The SMA and pre-SMA are considered part of the externally directed DAN (Vincent et al., 2008), but they are active during meditation along with the salience detecting/executive FPC network (Brefczynski-Lewis et al., 2007; Manna et al., 2010; Hasenkamp et al., 2012). The length of practice time of meditators, interestingly, was negatively correlated with all of these areas during the shifting phase, indicating that more meditation experience is associated with less activity during the shifting phase (Hasenkamp et al., 2012).

## SPINAL MOTOR NEURONS

The supplementary eye fields, frontal eye fields, pre-motor and motor cortices all share reciprocal connections with the LC (Shook et al., 1990). The LC projects extensively to the spinal cord depolarizing and enhancing spinal motor neuron excitability (Fung et al., 1991; White et al., 1991), making them receptive to the initiation of movement, while exercise, itself, spontaneously activates the LC (Warren et al., 1984; Haxhiu et al., 2003). These capacities of the LC may play a role in the attentive enhancement of movement during yoga.

## LC BEHAVIOR PATTERNS

### TONIC BASELINE FIRING

Higher baseline (tonic) firing in the LC is associated with enhanced labile attention and modest burst firing to various low salience distracter stimuli. This enhanced receptivity to the stimuli of the present moment is thought to optimize learning in unknown novel environments (Aston-Jones and Cohen, 2005).

During one of the earliest western scientific studies of meditation, the Zen masters reported that during meditation they had “more clearly perceived each stimulus than in their ordinary awakening state” (Kasamatsu and Hirai, 1966). Long-term meditators have repeatedly been found to exhibit an enhanced receptivity to stimuli (Jha et al., 2007; Slagter et al., 2007, 2009; Kerr et al., 2008; MacLean et al., 2010; Naranjo and Schmidt, 2012; Cahn et al., 2013; Mirams et al., 2013), including to low salience and habituated standard stimuli (Cahn et al., 2013), suggestive of higher tonic baseline LC activity.

Increased receptivity to stimuli, as initiated by the activation of the FPC orienting system to oddball stimuli, causes a subjective expansion of time (Tse et al., 2004). Such a subjective expansion of time has recently been observed in meditators (Kramer et al., 2013).

### PHASIC BASELINE FIRING

In contrast, lower, intermediate, more synchronous phasic baseline firing in the LC promotes larger, robust bursts to attended significant known stimuli, rapid well-learned dominant, autoassociative responses, enhanced selective attention, and reduced responding to distractor stimuli. This pattern is thought to optimize behavior in known familiar environments (Aston-Jones and Cohen, 2005) with a well-known coping response available.

### SNS MODULATION OF LC BASELINE FIRING

In the LC, changes in baseline firing, along with their associated subsequent bursting patterns, differentially modulate the state of the brain, the central nervous system, to optimize behavior in either novel or familiar environments, particularly if they are stressful environments.

Similarly, in the peripheral SNS, epinephrine (P-E) and P-NE are differentially released from the adrenal medulla (Mason et al., 1961; Brady, 1967; Frankenhaeuser, 1971; Stoddard et al., 1987; Morrison and Cao, 2000) under stress. Although they do not cross the blood brain barrier, P-E (Holdefer and Jensen, 1987) and P-NE (Svensson et al., 1980; Elam et al., 1984) inversely modify baseline firing in the LC.

During circumstances of novel stress a substantial amount of P-E is released from the adrenal medulla along with a small amount of P-NE (Mason et al., 1961; Brady, 1967; Frankenhaeuser et al., 1968; Frankenhaeuser, 1971). P-E elevates the baseline tonic firing in the LC (Holdefer and Jensen, 1987). This increases the release of C-NE, causing increased cortical arousal, decreased selective attention, and increased receptivity to the novel stimuli of the present moment. By the elevating of tonic baseline firing in the LC, P-E also enhances memory (Holdefer and Jensen, 1987) for the novel events through the increase in C-NE (Lemon et al., 2009; Reid and Harley, 2010).

In contrast to novel stress, under conditions of familiar stress (Mason et al., 1961; Brady, 1967; Frankenhaeuser et al., 1968; McCarty and Kopin, 1978; McCarty et al., 1978), especially with a well-known coping response available (Mandler, 1967; Frankenhaeuser et al., 1968), substantial amounts of P-NE are released from the adrenal medulla with virtually no P-E (Brady, 1967; Frankenhaeuser and Rissler, 1970; Frankenhaeuser, 1971). By elevating blood pressure, P-NE dose-dependently inhibits baseline tonic firing in the LC via the vagus and the NTS (Svensson et al., 1980; Elam et al., 1984). This causes rapid well-known dominant, autoassociative coping responses, increased selective attention, decreased responding to low salience distractor stimuli, and increased resistance to extinction (Craigmyle, N. A., unpublished data). Decreased perception of affect has also been observed (McCubbin et al., 2011). This classic stress and arousal behavior pattern (Easterbrook, 1959; Friedman et al., 1960, 1975; Zajonc, 1965; Eysenck, 1976; Geen, 1976; Geen and Gange, 1977) is consistently associated with relatively low, intermediate, phasic baseline LC activity (Aston-Jones and Cohen, 2005).

## INVERSE RELATIONSHIP OF CORTICAL AND PERIPHERAL SNS AROUSAL

As mentioned, there is an inverse relationship between cortical arousal and peripheral SNS arousal (Nagai et al., 2004a, b, 2009; Duschek et al., 2007, 2013). The contingent negative variation (CNV) has been used as an index of cortical arousal during orienting and attention, while changes in skin conductance were measured and pharmacological and biofeedback methods were used to elevate blood pressure, a measure of peripheral SNS arousal. Elevated blood pressure was shown to decrease the CNV amplitude in normotensive subjects (Duschek et al., 2007, 2013).

The dose-dependent inhibition of baseline firing in the LC by elevated blood pressure (Svensson et al., 1980; Elam et al., 1984) may contribute to this inverse correlation of peripheral SNS and cortical arousal.

In contrast, enhanced CNV cortical arousal-related activity in the AC, the midcingulate/SMA and the insula is associated with decreases in peripheral SNS arousal (Nagai et al., 2004b), which may be due to the capacity of these areas of the brain to inhibit the rostral VLM (Viltart et al., 2003), causing a reflex fall in SNS nerve activity and blood pressure (Standish et al., 1995). This relationship may also contribute to the inverse relationship of cortical and peripheral SNS arousal seen during meditation.

## PRINCIPAL AREAS MODULATING THE LC AND SNS – DIRECTLY AND RECIPROCALLY

A limited number of areas differentially influence activity in the integrated central noradrenergic LC and the peripheral noradrenergic SNS systems.

### AC, SMA, Pre-SMA

These areas have been briefly addressed above.

### ORBITOFRONTAL CORTEX

The orbitofrontal cortex (OFC) is another prominent descending cortical projection to the LC (Aston-Jones and Cohen, 2005). Activity in the AC and OFC is negatively correlated and they have complementary and reciprocal roles in monitoring the outcome of behavior (Aston-Jones and Cohen, 2005). While the AC is active in relation to self-generated decision-making, the OFC is active when the decisions are guided by the experimenter (Ullsperger and von Cramon, 2004; Walton et al., 2004), when the decisions and the reward characteristics of the stimuli are predictable (Baxter and Croxson, 2013; Rudebeck et al., 2013), are essentially known, and a coping response is available. Whereas the activity in the AC during salience detection and decision-making elevates tonic baseline firing in the LC enhancing receptivity to the various stimuli of the present moment, activity in the OFC may lower baseline firing in the LC potentiating selective attention and the rapid performance of expected well-known, autoassociative responses.

### ASCENDING AUTONOMIC TRACTS

The ascending autonomic tracts from the periphery also influence the LC. Cardiorespiratory, visceral and somatosensory autonomic stimuli regulate LC activity through ascending autonomic tracts with putative implications for psychiatry and psychopharmacology (Svensson, 1987). The cardiovascular stimuli from the autonomic environment seem to predominate over external environmental stimuli with respect to the LC’s influence on behavior (Svensson, 1987).

The parasympathetic vagus nerve influences activity in the LC, primarily via the NTS, while the ascending sympathetic lamina 1 STT projects directly and reciprocally to the LC (Craig, 1992).

### CENTRAL AUTONOMIC NUCLEI

The LC projects reciprocally to central autonomic nuclei including the sympathetic VLM, the parasympathetic DM, and regions of the NTS. The LC also projects to the region of the parasympathetic NA (Sakai et al., 1977; Westlund and Coulter, 1980). The LC core has been found to project extensively to regions giving rise to parasympathetic outflow, while the subcoeruleus (peri-coeruleus) region projects to sympathetic regions (Westlund and Coulter, 1980). The LC is a distinct part of the central neuronal circuit innervating various regions of the rat heart (Standish et al., 1995).

As mentioned, the NA contains primary source nuclei for the cardiopulmonary branch of the vagus (Standish et al., 1995), has myelinated axons thought to rapidly modulate the function of the heart and lungs (Standish et al., 1995) and some of its neurons simultaneously innervate both the adrenal gland and the stellate sympathetic ganglion (Jansen et al., 1995) through which it can rapidly modulate the SNS.

There are alpha 1 (Boychuk et al., 2012), alpha 2 (Haxhiu et al., 2003), and beta 1 (Bateman et al., 2012) adrenergic receptors in the NA, each differentially modulating NA function. Alpha 1 receptors facilitate inhibitory neurotransmission (Boychuk et al., 2012), while beta 1 adrenergic receptors decrease both inhibitory and excitatory neurotransmission to cardiac vagal neurons (Bateman et al., 2012).

Locus coeruleus activation dilates the airways via NA alpha 2 adrenergic receptors (Haxhiu et al., 2003), while fear and emotional distress may facilitate bronchoconstrictive attacks (Lehrer et al., 1993; Haxhiu et al., 2003; Rosenkranz et al., 2005, 2012). Exercise spontaneously activates the LC (Haxhiu et al., 2003) and dilates the airways (Warren et al., 1984; Haxhiu et al., 2003). The dilation of the airways by the LC via the NA may contribute to the decreased respiratory rate and HF-HRV, which are associated with meditation (Lazar et al., 2005; Tang et al., 2009; Kodituwakku et al., 2012) and yoga. The AC appears to be a critical component in the circuitry that links the development of peripheral symptoms with emotion and cognition in asthma (Rosenkranz and Davidson, 2009). Activation of the LC by the AC may play a role in the beneficial effects of mind–body influences in asthma.

The NA is also, as previously mentioned, closely linked to the rapid expression and regulation of emotional state (Porges, 1995; Tonhajzerova et al., 2013). A withdrawal of the cardiopulmonary vagal efferent outflow from the NA is seen during both acute and chronic stress, which, in healthy individuals, is accompanied by increased sympathetic tone (Porges, 1995), by increased levels of P-NE.

Following bilateral lesions of the LC, animals fail to show normal cardioaccelerator responses to threatening stimuli (Redmond, 1977; Snyder et al., 1977). The loss of the inhibitory influence of the LC on the NA, through its adrenergic receptors, may contribute to this failure.

### AMYGDALA

#### The AM is of particular importance in influencing the LC, SNS, and the AC

The AM forms the core of a second, early-activated emotional salience detecting network or orienting system comprised of the superior colliculus, pulvinar, and AM (Liddell et al., 2005; Luo et al., 2007; Tamietto and de Gelder, 2010; Van den Stock et al., 2011; de Gelder et al., 2012). This system acts as a pre-consciousness early warning system (Liddell et al., 2005; Luo et al., 2007; Tamietto and de Gelder, 2010; Van den Stock et al., 2011; de Gelder et al., 2012), particularly to threatening emotional stimuli, including threatening subliminal stimuli (Liddell et al., 2005). An early activation of the LC following subliminal stimuli has been observed, indicating this AM system may initially activate the LC (Liddell et al., 2005).

The early AM and the later cortical salience detecting orienting networks are reciprocally interconnected, sequentially activated, and both modulate the activity levels in the LC and in the SNS, each contributing differentially to adapt the state of the whole organism to environmental change.

The AM has reciprocal projections to the LC, the AC, and to the NTS and DM, amongst various other regions including the VLM, insula, and OFC (Price and Amaral, 1981; Amaral and Price, 1984; Volz et al., 1990).

The AM projects reciprocally to both the dorsal AC of the dmPFC and to the ventral AC of the vmPFC. The tract between the AM and the ventral AC is a white matter tract (Kim and Whalen, 2009), allowing for rapid transmission. Increased activity in the vmPFC is correlated with increased parasympathetic vagal activity (Lane et al., 2009; Tang et al., 2009) and the vmPFC is thought to exert a tonic influence on the parasympathetic NA during the resting state (Wong et al., 2007). Both the AM and the AC, independently, are required for the occurrence of conditioned bradycardia, the conditioned heart rate decrease that develops in response to significant stimuli (Powell et al., 1997).

Although the AM was long presumed to project to the NA (Schwaber et al., 1982; Volz et al., 1990) to rapidly activate the SNS (Porges, 1995), more recently, this could not be confirmed (Standish et al., 1995). The AM may, however, rapidly modulate activity in the NA through its white matter tract with the vmPFC.

Following stimuli, the AM directly receives an early relatively “crude” version of the stimuli via the superior colliculus and pulvinar in10–20 ms (Luo et al., 2007; Van den Stock et al., 2011). If the stimulus is significant, the AM responds rapidly within 20–30 ms and activates the LC (Liddell et al., 2005), in another10–20 ms (Bouret et al., 2003). These activations occur before the cortical frontal eye fields are activated at approximately 64 ms, the supplementary eye field at 81 ms, and the AC at100 ms (Pouget et al., 2005). All the above activations appear to commence prior to conscious awareness of the stimuli (Fried et al., 2011; Kreiman, 2012).

When conditions allow for cortical processing, the mPFC, both dorsal and ventral, is believed to regulate and control the AM, either increasing or decreasing AM activity (Ohman, 2005; Kim et al., 2011a, b). During rest, in normal low anxiety humans, activity in the vmPFC is positively correlated with the AM, while dmPFC activity is negatively correlated (Kim et al., 2011b).

Following a familiar, stressful, significant stimulus, with a known coping response available, the AM is thought to “take the PFC “off line” to allow faster, more habitual responses mediated by the posterior and/or subcortical structures to regulate behavior” (Arnsten, 1997).

The cortical mPFC, however, can in turn regulate and control the AM (Ohman, 2005; Kim et al., 2011a, 2011b), to either enhance activity in the AM or to take the AM, itself, “off line.”

Meditators exhibit lower activity in the AM (Brefczynski-Lewis et al., 2007; Creswell et al., 2007; Lutz et al., 2008), an inhibition of the AM occurs during meditation (Lutz et al., 2008) and decreased gray matter density in the AM emerges over time (Hölzel et al., 2010).

Arnsten (1997) has suggested the AM may take the PFC “off line” by activating the LC, causing high levels of C-NE and dopamine in the cortex to inhibit the PFC – the DLPFC in particular. Interestingly, evidence points to the LC as a common origin for both the C-NE and the dopamine in the cortex (Devoto and Flore, 2006).

The central nucleus of the AM (CeA) projects to the LC. Stimulation of the CeA stimulates the LC, producing a large single or double burst, followed by an extended refractory period (Bouret et al., 2003). In response to a familiar, stressful, significant stimulus, the CeA would be expected to stimulate the LC, eliciting a large burst of C-NE, followed by an extended refractory period, SNS peripheral arousal and elevated blood pressure, resulting in decreased cortical arousal (Duschek et al., 2007, 2013).

#### The LC projects to the basolateral amygdala influencing memory

Activation of the LC modulates activity in the basolateral amygdala (BLA) by releasing C-NE. The C-NE inhibits spontaneous firing in the majority of the BLA neurons via alpha 2 adrenergic receptors, while exciting others via beta adrenergic receptors (Buffalari and Grace, 2007).

Elevated activity in the LC acts via the BLA beta adrenergic receptors to enhance the consolidation of memory (Roozendaal and McGaugh, 2011) in the hippocampus (McReynolds et al., 2010), and the cortex (Chavez et al., 2013), in both the mPFC (Roozendaal et al., 2009) and in the insula (Bermudez-Rattoni et al., 2005).

Arousal-induced release, or systemic injection of the peripheral adrenal hormones P-E and cortisol (corticosterone in rats) enhances the consolidation of memory via the action of C-NE on BLA beta receptors (Chavez et al., 2013).

Considerable evidence indicates that peripheral action on the vagus nerve stimulates the LC to release this C-NE in the BLA (McIntyre et al., 2012). Systemic administration of P-E has been shown to dose-dependently elevate tonic baseline firing in the LC (Holdefer and Jensen, 1987). In contrast, amphetamines, like P-NE (Svensson et al., 1980; Elam et al., 1984), inhibit LC baseline firing (Holdefer and Jensen, 1987).

Activation of the LC by exercise has also been shown to enhance memory, including in older people and those with the early stages of Alzheimer’s disease (Segal et al., 2012).

Following acute stress there is a period of increased connectivity between the AC, LC, and AM, which may contribute to the consolidation of memory for the significant events (van Marle et al., 2010). The LC is active during slow wave sleep contributing to the consolidation and the re-consolidation of memory (Sara, 2010; Eschenko et al., 2012) and is also involved in the successful retrieval of emotional memory (Sterpenich et al., 2006).

#### Long-term stress induces homeostatic changes in the interrelationship between the AM and the LC

In controls, increased norepinephrine in the BLA inhibited spontaneous firing in the majority of the BLA neurons, with some showing excitation at lower doses but inhibition at higher doses. Norepinephrine also decreased responsiveness of these neurons to electrical stimulation of the entorhinal and sensory association cortices (Buffalari and Grace, 2009). However, following chronic cold stress, norepinephrine led to an increase in the excitatory effects of norepinephrine on BLA neurons and a facilitation of responses to the stimulation of the entorhinal and sensory association cortices (Buffalari and Grace, 2009).

During stress, the CeA is a major source of elevated corticotropin-releasing factor (CRF) in the peri-coerulear LC (Van Bockstaele et al., 2001, 2010), the area of the LC that projects to sympathetic regions (Westlund and Coulter, 1980). CRF activates the LC raising C-NE levels in the posterior parts of the ventral medial (VPm) thalamus (Devilbiss et al., 2012). Although increased levels of LC output can facilitate sensory-evoked responses of VPm thalamic and barrel field cortical neurons in an inverted U dose–response relationship, high levels of peri-coerulear LC infusions of CRF caused a dose-dependent suppression of sensory-evoked discharge in the VPm thalamus and in cortical barrel field neurons resulting in a net decrease in signal-to-noise of sensory-evoked responses (Devilbiss et al., 2012).

## PAIN AND THE COERULEOSPINAL CENTRIFUGAL PAIN CONTROL SYSTEM

The Vpm thalamus is the thalamic relay nucleus for the sympathetic STT, which carries the afferent interoceptive information, including pain, to the anterior insula (by way of the dorsal posterior insula) and to the somatosensory BA3a (Craig, 2004, 2009). The branch of the STT that projects to the AC relays in the ventro-caudal portion of the medial dorsal thalamic nucleus (Craig, 2004).

The LC projects reciprocally to the STT (Craig, 1992; Westlund and Craig, 1996). The descending coeruleospinal inhibitory pathway from the LC and subcoeruleus (peri-coeruleus) is one of the centrifugal pain control systems. The function of this LC system is thought to maintain the accuracy of intensity coding in the dorsal horn, while inhibiting nocioceptive signals in order to extract other sensory information that is essential for circumstantial judgment (Tsuruoka et al., 2011).

Activation of the LC can produce profound antinociception (Tsuruoka et al., 2011, 2012; Hayashi et al., 2012) and can inhibit the nociceptive activity of spinal dorsal horn neurons and trigeminal subnucleus caudalis neurons (Hayashi et al., 2012). Nocioceptive signals from visceral organs and cutaneous receptive fields converge on single dorsal horn neurons. Electrical stimulation of the LC inhibited both the visceral (colorectal distention) and the cutaneous pinch responses, with a reduction in the intensity-response magnitude curve without a change in the response threshold (Hayashi et al., 2012).

Fear-induced antinociception also occurs via LC pathways (Biagioni et al., 2013), seizure-induced antinociception involves LC alpha 2 and beta adrenergic receptors (Felippotti et al., 2011) and the LC is thought to play a role in the antinociception caused by stimulation of the motor cortex (Viisanen and Pertovaara, 2010).

Chronic pain stress changes the influence of the AM on the LC from excitation to inhibition (Viisanen and Pertovaara, 2007).

Locus coeruleus responses to noxious stimulation were initially enhanced following experimental neuropathy, however, after 10–14 days microinjections of glutamate into the CeA produced a dose-related inhibition of the discharge rate of LC neurons. There was no significant effect on discharge rates in control groups. Spinal antinociception due to LC electrical stimulation was also weaker in the nerve injured rats. The enhanced inhibition of the LC by the CeA was thought to suppress the noradrenergic pain inhibition and promote neuropathic pain (Viisanen and Pertovaara, 2007).

Long-term pain can increase the likelihood of mood or anxiety disorders by as much as threefold (The Neuroscientist, 2012).

Long-term chronic pain (>28 days) caused an increase in LC bursting activity, tyrosine hydroxylase expression and that of the norepinephrine transporter; and enhanced expression and sensitivity of the inhibitory alpha 2 adrenoceptors in the LC. This was accompanied by an inability to cope with stressful situations, depressive and anxiogenic-like behaviors (Alba-Delgado et al., 2013). As mentioned, increased LC bursting activity is associated with lower, inhibited, LC baseline levels.

Meditation has been repeatedly found to reduce pain (Zeidan et al., 2012).

In a 2012 review of meditation-related pain relief, mindfulness meditation was found to significantly reduce pain through a number of unique brain mechanisms (Zeidan et al., 2012). In meditators during pain, higher activation was seen in the dorsal AC and insula, but was reduced in the medial prefrontal-OFC (vmPFC), DLPFC, and AM. Meditation significantly reduced lower level processing in the primary somatosensory cortex, while the increased activity in the rostral AC and anterior insula was associated with intensity reductions and the decreased orbitofrontal and thalamic activity was associated with reduced unpleasantness (Zeidan et al., 2012).

In meditators, most of the areas of the brain associated with the reduction of pain, including the salience detecting dorsal AC and insula, the primary somatosensory area and the thalamus, receive projections from the ascending STT (Craig, 2004, 2009). The reduced lower level processing in the primary somatosensory area, decreased thalamic activity and the intensity reductions associated with the higher dorsal AC and insula activity could be the result of the LC’s profound antinociceptive action on the ascending STT, reducing the response intensity magnitude curve without a change in response threshold (Hayashi et al., 2012). By this route, the AC activation of the LC may contribute to meditation-induced reduction of pain.

## VAGUS NERVE STIMULATION

Chemical or electrical stimulation of the vagus nerve alters LC activity and that of its forebrain targets suggesting that the therapeutic effects of vagal nerve stimulation (VNS) may involve the LC-noradrenergic system (George and Aston-Jones, 2010). It has been suggested that the effects of VNS on learning and memory, mood, seizure suppression, and recovery of function following brain damage are mediated, in part, by the release of C-NE in the terminal fields of the LC (Roosevelt et al., 2006). VNS is also being investigated with respect to anxiety (George et al., 2008), inflammation, and the immune response (George and Aston-Jones, 2010).

The initiation of VNS activates the LC (Dorr and Debonnel, 2006), increasing not only the spontaneous (baseline) firing rate, but also the percentage of LC neurons firing in bursts (Manta et al., 2009). The LC has an excitatory influence on the dorsal raphe (Dorr and Debonnel, 2006). Long-term (14 days) VNS increased activity in the serotonin neurons of the dorsal raphe nucleus, as seen with other antidepressant treatments (Manta et al., 2009), through an activation of alpha 1 adrenergic neurons and increased tonic activation of post-synaptic 5-HT1A receptors in the hippocampus (Manta et al., 2013). Long-term VNS, further, significantly increased extracellular C-NE levels in the prefrontal cortex and hippocampus and enhanced the tonic activation of post-synaptic alpha 2 adrenoceptors on pyramidal neurons (Manta et al., 2013).

A recent review discusses some of the beneficial effects of mindfulness-based stress reduction, mindfulness-based cognitive therapy, and Zen meditation to alleviate depression, anxiety, pain, and psychological distress (Marchand, 2012).

The kinds of pain and suffering alleviated by meditative practices, are remarkably similar to those alleviated by stimulation of the vagus. The increased activation of the LC by both practices may contribute to the beneficial similarities.

## BLOOD VOLUME, OXYGEN DEMAND, CELLULAR ENERGY METABOLISM, AND INFLAMMATION

The LC–NE network optimizes coupling of cerebral blood volume with oxygen demand through local vasodilation in active brain areas, while constricting volume in other areas (Bekar et al., 2012). Of increasing interest is the modulation by C-NE of glia, astrocytes, oligodendrocytes, and microglia in their critical support functions (Bekar et al., 2008; Chandley and Ordway, 2012; O’Donnell et al., 2012). C-NE, for example, acts on astrocytes to enhance glutamate uptake, while increasing production and breakdown of glycogen (O’Donnell et al., 2012). Microglia, often thought of as the primary immune effector cells of the CNS, represent a major target of C-NE signaling in the cortex (O’Donnell et al., 2012). C-NE modulates microglia disease responses, suppressing inflammatory gene transcription and reducing expression of pro-inflammatory cytokines, while enhancing production of brain-derived neurotrophic factor (BDNF) to promote neuronal survival (O’Donnell et al., 2012). The mechanism by which C-NE reduces the expression of pro-inflammatory cytokines is still a topic of debate, but may involve the regulation of the NF-kB signaling system by B2-adrenergic receptor driven increases in cAMP (O’Donnell et al., 2012).

Yogic meditation downregulated transcripts of pro-inflammat-ory cytokines, decreasing expression of NF-kB associated pro-inflammatory genes (Black et al., 2013). Mindfulness-based stress reduction training also resulted in a significantly smaller post-stress inflammatory response (Rosenkranz et al., 2013). The regulation of gene expression by yoga, meditation, and related practices has recently begun to be investigated (Saatcioglu, 2013). The activation of the LC, by the AC and by exercise, may again be a contributing factor.

## NEUROPLASTICITY

In post-mortem studies of depressed humans a loss of glial cells has been demonstrated in the AC, DLPFC, and OFC, amongst other areas (Chandley and Ordway, 2012). It has been hypothesized that given the intimate functional relationship between C-NE and glia, particularly astrocytes, the glial deficits may be secondary to a deficiency of C-NE (Chandley and Ordway, 2012). Humans with dopamine-B-hydroxylase deficiency, who have no norepinephrine, exhibit a smaller total brain volume (Jepma et al., 2011).

Research indicates that LC neuron loss appears with aging (Shibata et al., 2006) and depression (Shibata et al., 2007), and that such loss is prominent in Parkinson’s and Alzheimer diseases (Bekar et al., 2012). A diminished ability to couple blood volume to oxygen demand (Bekar et al., 2012), and to support other neuronal and non-neuronal cellular requirements (Bekar et al., 2008; Chandley and Ordway, 2012; O’Donnell et al., 2012) due to a reduction in C-NE from LC neurons, may contribute to their pathogenesis.

Structural changes are observed in various brain areas of meditators (Lazar et al., 2005; Hölzel et al., 2010; Tang et al., 2010, 2012; Luders et al., 2011, 2013; Grant et al., 2013; Kang et al., 2013; Luders, 2013). The elevated C-NE due to the activation of the LC by the AC may play a role.

## DISCUSSION

In early 2012, when the Hasenkamp paper was published and research for this paper began, some aspects of the AC’s activation of the LC and inhibition of the SNS to maximize adaptation in changing environments were already known. Included were the increased receptivity to the stimuli of the present moment caused by activating the LC; the possible reduction of stress through the inhibition of the stress associated SNS; the LC behavior patterns and the potential importance of understanding that the LC is not activated in tandem with the SNS, but that stress associated elevated P-NE inhibits the LC dose-dependently enhancing the phasic behavior pattern; that higher tonic base line firing is not associated only with stress and arousal, but with increased receptivity to the stimuli of the present moment, with increased awareness. These aspects were already understood and seemed of significant importance to understanding the neuroscientific process underlying the changes in state initiated by meditation.

During 2012 and 2013, however, numerous papers have been published that further implicate the integrated norepinephrine systems in the enhancement of cortical executive network functions and in the modulation of the AM, NA, VPm, STT, and pain, as well as in the modulation of astrocytes and glia, blood volume, oxygen supply, cellular energy metabolism, inflammation, and even of neuroplasticity.

These aspects were generally unknown in early 2012, and have vastly expanded the understanding of the role of the AC’s activation of the LC and inhibition of the SNS in general, and potentially in the beneficial changes initiated by meditation.

The AC and the anterior insula, together, form the cortical salience detecting network, are usually jointly activated, contain numerous recently evolved von Economo neurons, and undergo structural changes in longer term meditators. Reviews of their functions (Craig, 2009; Medford and Critchley, 2010; Menon and Uddin, 2010) implicate them in the capacity for awareness of self and awareness of the moment.

The AC is active during salience detecting and monitoring of the stimuli in the present moment, is naturally activated by novel or significant stimuli and normally ceases activity as the event-encoding cycle ends. During mindfulness meditation the AC is active “at the moment of awareness of mind wandering.” This suggests the AC is active “at the moment of awareness” of whatever is occurring now, in the present moment.

During meditation, following the initial “awareness of mind wandering,” one “shifts” to “sustained attention,” one shifts to sustain the attention of awareness of the present moment.

Without the development of a capacity to intentionally sustain the attention of awareness of the present moment, this quality of attention will normally be lost as the event-encoding cycle ends. The various practices of meditation, however, can develop a capacity to intentionally sustain the attention of awareness of whatever is occurring in the present moment, irrespective of the nature of the stimuli and irrespective of the environment from which they come, whether external, internal, or interoceptive.

As mentioned, the salience detecting/executive FPC cortical orienting network, including both the AC and the insula, shifts between the external DAN and the internal DMN, while the AC and insula receive information from the interoceptive STT. This may allow the AC, in coordination with the insula, to monitor and detect the salience of the stimuli of the present moment from all environments, irrespective of the nature of the stimuli, whether known or Unknown.

Meditation may develop the capacity for an intentional attention of awareness, which activates the AC of the salience detecting/executive FPC cortical orienting network, initiating a variety of physiological cascades through its modulation of the central LC and peripheral SNS norepinephrine systems, via both parasympathetic and sympathetic routes, to maximize receptivity and adaptation in changing environments.

Buddha realized he had found a pathway to the elimination of pain and suffering. The inhibition of the SNS and activation of the LC by the AC during meditation may be a contributing factor worthy of further scientific exploration.

## Conflict of Interest Statement

The author declares that the research was conducted in the absence of any commercial or financial relationships that could be construed as a potential conflict of interest.
